# Remarks on Granular Conjunctiva

**Published:** 1855-04

**Authors:** 


We extract the following practical remarks on Granular Conjunctiva, from the report of a clinical lecture by Prof. E. M. Moore, of the Starling Medical College, published in the Ohio Medical Journal:

"In speaking of the various persons who came before the class with this disease, Dr. Moore took occasion to call attention to the present tendency to underrate the advantages of local depletion. Scarification of the lids had been very much reprobated for producing permanent ridges upon the tarsus, which maintained the inflammation upon the ball, and which remained permanent. 'This,' he remarked, 'it seems to me, need not happen, and if the sharp knife is decidedly used, so that the incision does not go deeper than the mucous membrane, the roughness will not occur. The scarification alone, even as a local measure, cannot be relied on. Nitrate of silver, and other caustics, must be employed to give smoothness to a surface which is roughened by organized effusions. In the application of these remedies, and especially of the nitrate, great care is to be used, in order to avoid an excessive quantity at any one time. Violent, and even dangerous, inflammation often results from neglect of proper precaution. Others propose a strong solution of 20 or 40 grains to the ounce, which is applied by camel's hair pencil. Here we get too much of the solution, not strong enough. The best mode I have ever seen is to moisten a brush, and leave not a single drop upon its end. This now rubbed upon a crystal of nitrate of silver, gives us an exceedingly minute quantity of saturated solution. If the brush is drawn across the granulated lid, an extremely delicate, but perfect, pelicle is formed on the surface, which subserves the double purpose in destroying the sharp points of the granules and protecting the eye from their attrition. This is to be frequently repeated, often daily. But you must not understand that local measures alone are sufficient to the cure of this disease. The general health must be carefully attended to, for, like other chronic inflammation, it is almost hopeless to expect recovery unless this is placed upon a satisfactory footing.''

